# A Proteomic Approach to Uncover Neuroprotective Mechanisms of Oleocanthal against Oxidative Stress

**DOI:** 10.3390/ijms19082329

**Published:** 2018-08-08

**Authors:** Laura Giusti, Cristina Angeloni, Maria Cristina Barbalace, Serena Lacerenza, Federica Ciregia, Maurizio Ronci, Andrea Urbani, Clementina Manera, Maria Digiacomo, Marco Macchia, Maria Rosa Mazzoni, Antonio Lucacchini, Silvana Hrelia

**Affiliations:** 1 Department of Clinical and Experimental Medicine, University of Pisa, 56126 Pisa, Italy; laura.giusti@unipi.it (L.G.); antonio.lucacchini@gmail.com (A.L.); 2 School of Pharmacy, University of Camerino, 62032 Camerino, Italy; cristina.angeloni@unicam.it; 3 Department for Life Quality Studies, Alma Mater Studiorum, University of Bologna, 47921 Rimini, Italy; maria.barbalace2@unibo.it; 4 Department of Pharmacy, University of Pisa, 56126 Pisa, Italy; serelac@hotmail.it (S.L.); clementina.manera@unipi.it (C.M.); maria.digiacomo@unipi.it (M.D.); marco.macchia@unipi.it (M.M.); maria.mazzoni@unipi.it (M.R.M.); 5 Department of Rheumatology, GIGA Research, Centre Hospitalier Universitaire (CHU) de Liège, University of Liège, 4000 Liège, Belgium; ciregia@gmail.com; 6 Department of Medical, Oral and Biotechnological Sciences, University G. d’Annunzio of Chieti-Pescara, 65127 Pescara, Italy; maurizio.ronci@unich.it; 7 Institute of Biochemistry and Clinical Biochemistry, Catholic University, 00198 Rome, Italy; andrea.urbani@unicatt.it

**Keywords:** proteomics, oleocanthal, oxidative stress, neurodegeneration, heat shock proteins, peroxiredoxins, proteasome

## Abstract

Neurodegenerative diseases represent a heterogeneous group of disorders that share common features like abnormal protein aggregation, perturbed Ca^2+^ homeostasis, excitotoxicity, impairment of mitochondrial functions, apoptosis, inflammation, and oxidative stress. Despite recent advances in the research of biomarkers, early diagnosis, and pharmacotherapy, there are no treatments that can halt the progression of these age-associated neurodegenerative diseases. Numerous epidemiological studies indicate that long-term intake of a Mediterranean diet, characterized by a high consumption of extra virgin olive oil, correlates with better cognition in aged populations. Olive oil phenolic compounds have been demonstrated to have different biological activities like antioxidant, antithrombotic, and anti-inflammatory activities. Oleocanthal, a phenolic component of extra virgin olive oil, is getting more and more scientific attention due to its interesting biological activities. The aim of this research was to characterize the neuroprotective effects of oleocanthal against H_2_O_2_-induced oxidative stress in neuron-like SH-SY5Y cells. Moreover, protein expression profiling, combined with pathways analyses, was used to investigate the molecular events related to the protective effects. Oleocanthal was demonstrated to counteract oxidative stress, increasing cell viability, reducing reactive oxygen species (ROS) production, and increasing reduced glutathione (GSH) intracellular level. Proteomic analysis revealed that oleocanthal significantly modulates 19 proteins in the presence of H_2_O_2_. In particular, oleocanthal up-regulated proteins related to the proteasome, the chaperone heat shock protein 90, the glycolytic enzyme pyruvate kinase, and the antioxidant enzyme peroxiredoxin 1. Moreover, oleocanthal protection seems to be mediated by Akt activation. These data offer new insights into the molecular mechanisms behind oleocanthal protection against oxidative stress.

## 1. Introduction

With the acceleration of the size of the aging population, the world is now experiencing the “aging era” characterized by an increased incidence of neurodegenerative diseases like Alzheimer’s disease (AD) and Parkinson’s disease (PD) [1]. Neurodegenerative diseases represent a heterogeneous group of disorders whose common characteristic is the progressive and selective loss of neurons. Although many studies have been carried out to understand the pathophysiology of neurodegenerative diseases over the years, more efforts are still needed to uncover the processes that trigger these disorders. Despite different aetiologies, neurodegenerative disorders share common features like abnormal protein aggregation, perturbed Ca^2+^ homeostasis, excitotoxicity, impairment of mitochondrial functions, apoptosis, inflammation, and oxidative stress [2,3]. Oxidative stress is an imbalance between accumulation and removal of reactive oxygen species (ROS). The brain is especially susceptible to oxidative stress due to its high oxygen consumption, high content of oxidizable polyunsaturated fatty acids, and low level of endogenous antioxidants and antioxidant enzymes [4,5,6]. It has been shown that oxidative stress is involved in the onset and progression of neurodegenerative diseases like PD [2,7,8,9,10] and AD [11,12,13].

Despite recent advances in the research of biomarkers, early diagnosis, and pharmacotherapy, there are no treatments that can halt the progression or reverse the brain changes of any age-associated neurodegenerative diseases. This is likely due to the multifactorial nature of these pathologies that arise from a confluence of multiple, toxic insults. 

Diet is considered one of the most important factors in lifestyle. Diet strongly influences the occurrence of cardiovascular and neurodegenerative diseases and, hence, a healthy lifestyle can be related to healthy aging [14]. Numerous epidemiological studies indicate that long-term intake of a Mediterranean diet, characterized by a high consumption of extra virgin olive oil, correlates with better cognition in aged populations [15,16,17]. Olive oil phenolic compounds have been demonstrated to possess different biological activities like antioxidant [18], antithrombotic [19], and anti-inflammatory [20] activities. Oleocanthal, a dialdehydic form of (−)-deacetoxyligstroside glycoside, is one of the phenolic components of extra virgin olive oil [21]. Even though oleocanthal makes up only 10% of the olive’s phenols, it is getting more and more scientific attention due to its interesting biological activities [22,23]. Oleocanthal is responsible for the bitter and pungent taste of extra virgin olive oil, and has anti-inflammatory properties similar to the nonsteroidal anti-inflammatory drug ibuprofen [24]. In vitro, it has been shown that oleocanthal is effective on the key mediators of AD pathogenesis, amyloid-β and hyper-phosphorylated tau proteins [25,26,27,28], which contribute significantly to neurodegeneration and memory loss [29]. Moreover, oleocanthal reduces astrocyte activation and interleukin-1β levels in vivo [21]. Studies on the antioxidant activities of oleocanthal are limited. Only one study carried out in isolated human monocytes showed that oleocanthal inhibits nicotinamide adenine dinucleotide phosphate oxidase (NOX) activity and reduces the intracellular level of superoxide anion [30].

The aim of this research was to characterize the neuroprotective effects of oleocanthal against H_2_O_2_-induced oxidative stress in the neuron-like SH-SY5Y cell line. Moreover, protein expression profiling, combined with pathways analyses, was used to investigate the molecular events related to the protective effects, and to gain insight into the underlying mechanisms of neuroprotection and oxidative damage.

## 2. Results

### 2.1. Neuroprotective Effects of Oleocanthal against H_2_O_2_-Induced Damage

**Figure 1:**
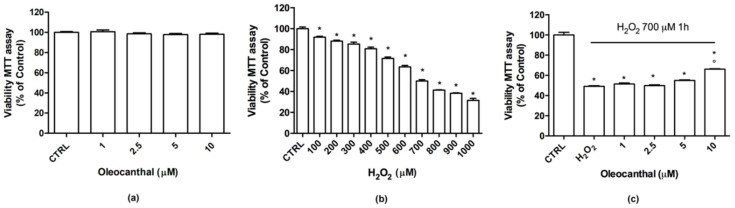
Viability of differentiated SH-SY5Y treated with oleocanthal in the absence/presence of H_2_O_2_. (**a**) Cells were treated with oleocanthal (1–10 µM) for 24 h, (**b**) cells were treated with peroxide (0.1–1 mM), or (**c**) cells were treated with oleocanthal (1–10 µM) and after 24 h exposed to 700 µM H_2_O_2_ for 1 h; cell viability was measured by MTT assay. Each bar represents means ± SEM of at least four independent experiments. Data were analyzed by one-way analysis of variance (ANOVA) followed by Bonferroni’s test. * *p* < 0.05 with respect to control (CTRL); ° *p* < 0.05 with respect to H_2_O_2_.

To investigate the potential cytotoxicity of oleocanthal, differentiated SH-SY5Y cells were treated with different concentrations (1–10 µM) of oleocanthal for 24 h (Figure 1a). Of note, oleocanthal did not show any effect on cell viability at any tested concentrations. To study the potential protective activity of oleocanthal against oxidative injury, cells were treated with increasing concentrations (1–10 µM) of oleocanthal before the induction of oxidative stress by 700 µM H_2_O_2_ exposure for 1 h (Figure 1c). This peroxide concentration has been chosen as it reduces cell viability by 50% with respect to control cells (Figure 1b). Moreover, similar H_2_O_2_ concentrations have been recently used by Piras et al. [31] in differentiated SH-SY5Y cells.

Cell viability was measured by 3-(4,5-dimethylthiazol-2-yl)-2,5diphenyl-tetrazolium bromide (MTT) assay. As expected, H_2_O_2_ significantly reduced cell viability with respect to control cells. Quantities of 1 to 5 µM oleocanthal were not able to counteract peroxide’s deleterious effects as cell viability was comparable to that of H_2_O_2_-treated cells. On the contrary, 10 µM oleocanthal significantly increased cell viability with respect to H_2_O_2_-treated cells. For these reasons, the subsequent experiments were carried out using 10 µM oleocanthal.

### 2.2. Antioxidant Activity of Oleocanthal against H_2_O_2_-Induced Oxidative Stress

**Figure 2:**
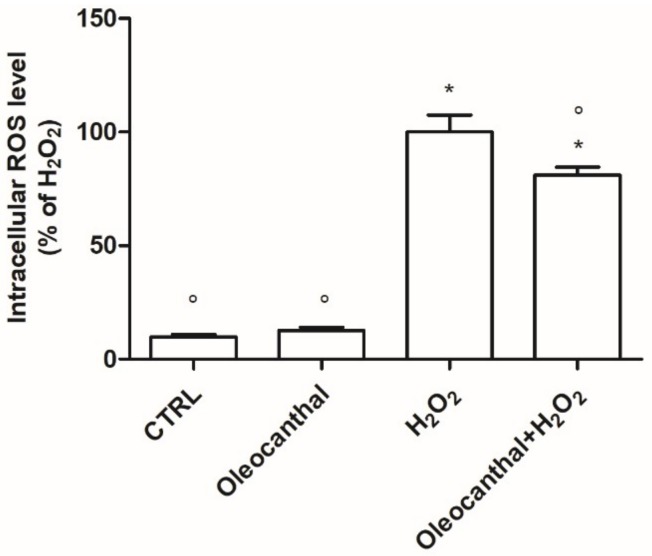
Antioxidant activity of oleocanthal against H_2_O_2_ in differentiated SH-SY5Y cells. Cells were treated with 10 μM oleocanthal and after 24 h were exposed to H_2_O_2_. Intracellular reactive oxygen species (ROS) levels were measured with the peroxide-sensitive probe DCFH-DA as reported in Materials and Methods. Data are expressed as a percentage with respect to H_2_O_2_-treated cells. Each bar represents mean ± SEM of at least four independent experiments. Data were analyzed by one-way ANOVA followed by Bonferroni’s test. * *p* < 0.05 with respect to CTRL; ° *p* < 0.05 with respect to H_2_O_2_.

The ability of oleocanthal to counteract H_2_O_2_-induced intracellular ROS production was investigated by the 2,7-dichlorodihydrofluorescein diacetate (DCFH-DA) assay. As illustrated in Figure 2, incubation of differentiated SH-SY5Y cells with H_2_O_2_ resulted in a significant and marked increase in intracellular ROS levels. Oleocanthal, in contrast, significantly reduced intracellular ROS levels compared to H_2_O_2_. There was no change in ROS levels after treatment with oleocanthal alone.

**Figure 3:**
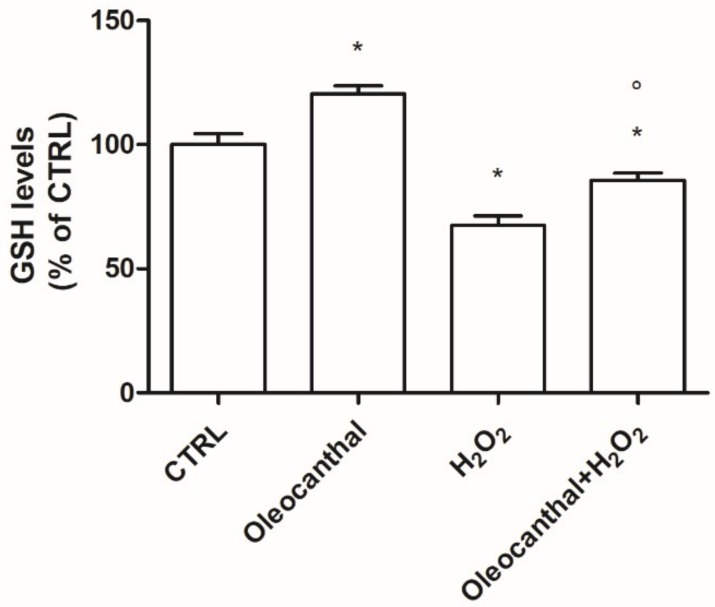
Effect of oleocanthal on GSH levels in differentiated SH-SY5Y cells. Cells were treated with 10 μM oleocanthal and after 24 h were exposed to peroxide. GSH levels were measured using the fluorescence probe MCB as reported in Materials and Methods. Each bar represents the mean ± SEM of four independent experiments. Data were analyzed by one-way ANOVA followed by Bonferroni’s test. * *p* < 0.05 with respect to CTRL; ° *p* < 0.05 with respect to H_2_O_2_.

As GSH is the most abundant endogenous antioxidant [32], we next evaluated the effect of oleocanthal treatment in the absence/presence of H_2_O_2_ on intracellular GSH levels by monochlorobimane (MCB) assay (Figure 3). Interestingly, oleocanthal alone was able to increase GSH levels compared with control cells. Exposure to H_2_O_2_ caused a significant decrease in GSH levels; meanwhile, pretreatment with oleocanthal significantly increased the amount of GSH compared with in H_2_O_2_-treated cells.

### 2.3. Protein Expression Analysis

**Figure 4:**
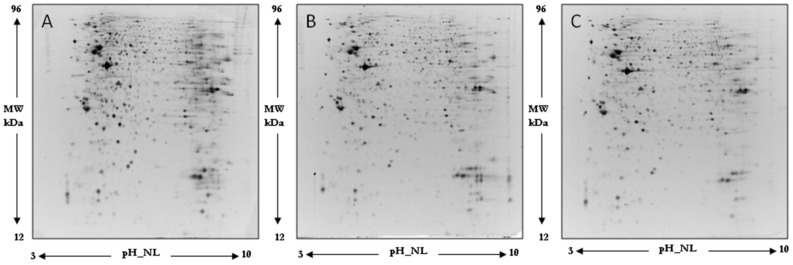
Representative 2D gel map of differentiated SH-SY5Y human neuroblastoma cellular protein extracts. (**A**) Control cells; (**B**) H_2_O_2_-treated cells; (**C**) Oleocanthal + H_2_O_2_ treated cells. Proteins were separated in a 3–10 nonlinear gradient. Sodium dodecyl sulfate polyacrylamide gel electrophoresis (SDS-PAGE) was performed at 12% of acrylamide.

Figure 4 illustrates a representative two-dimensional electrophoresis (2DE) image of differentiated SH-SY5Y human neuroblastoma cellular protein extracts. The quality of the gels was assessed by the software Same Spot which includes the SpotCheck function. SpotCheck is a separate quality control workflow which allowed us to objectively assess the quality of our gels images. We selected a “gold standard” gel (a reference gel) and each gel was tested against it; the software confirmed that we ran gels in a reproducible manner.

Overall, an average of 1100 ± 150 spots was found within a nonlinear pH range from 3 to 10.

Normalized spot volumes were analyzed by the ANOVA test to detect the proteins which were significantly (*p* < 0.05, *q* value < 0.05) differentially expressed among different comparisons. Regarding comparison with the control of the 46 spots detected as modified by the treatment with H_2_O_2_, 6 were up- and 40 down-regulated. Twelve spots were found differentially expressed in cells treated with oleocanthal; 5 were up- and 7 down-regulated. Finally, of the 46 spots significantly modified by the addition of oleocanthal + H_2_O_2_, 14 were up- and 32 down-regulated.

**Figure 5:**
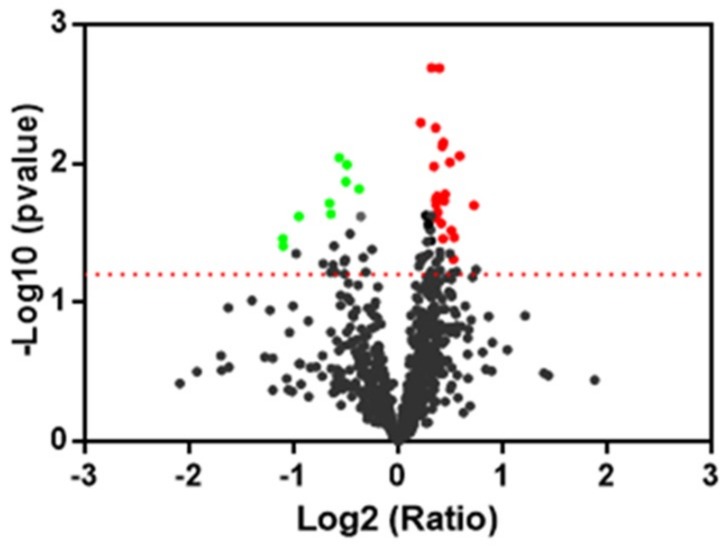
Protein expression profiling of oleocanthal + H_2_O_2_ vs. H_2_O_2_. Scatter plot of fold change (*x* axis), against log *p*-value (*y* axis) of all quantified proteins. Up- and Down-regulated proteins are colored red and green, respectively. Dotted line indicates the threshold of significance.

To investigate the protection of oleocanthal against the detrimental effect of H_2_O_2_, a comparison between oleocanthal + H_2_O_2_ vs. H_2_O_2_ was carried out. A volcano plot was constructed to represent fold change in protein expression (Figure 5). By this way, 31 spots were found with significant change of expression; 22 were up- and 9 down-regulated.

All spots of interest were selected and excised from the gel and identified by nano-LC-ESI-MS/MS.

A list of identified proteins, with molecular weight (MW), isoelectric point (pI), score, and coverage values of MS/MS is shown in Table 1, while Table 2 and Table 3 show the protein names, ratio, and *p* values of proteins resulting as significantly changed in the comparison between H_2_O_2_ vs. control and oleocanthal + H_2_O_2_ vs. H_2_O_2_, respectively.

### 2.4. Pathway and Network Analysis

**Figure 6:**
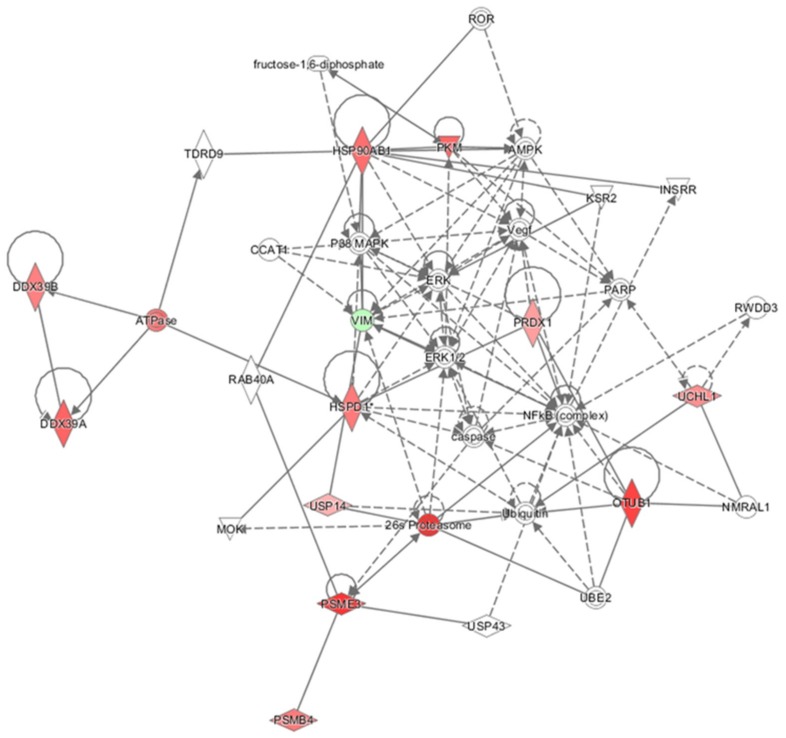
Functional Network. Proteins differentially expressed resulting from the oleocanthal + H_2_O_2_ vs. H_2_O_2_ comparison were functionally analyzed through the use of QIAGEN’s Ingenuity Pathway Analysis. Network describes functional relationships among proteins based on known associations in the literature. Solid line: direct interaction; dotted line: indirect interaction. * This protein has been identified in many spots.

For the oleocanthal + H_2_O_2_ vs. H_2_O_2_ comparison, proteins found differentially expressed were included in the Ingenuity Pathways Analysis (IPA) analysis to identify molecular and cellular functions and to investigate whether these proteins work together in specific networks. Therefore, the software generated a main network (Figure 6) with associated biofunctions “Cancer, Organismal Injury, and Abnormalities” and focused on canonical pathways which showed our differentially expressed molecules to fall into functional categories such as protein synthesis, protein degradation, cell death and survival, and cellular function and maintenance. All the proteins found to be up- and down-regulated may concur in an upstream regulator analysis to predict if transcription factors or genes could be activated or inactivated in agreement with the *z*-score value (*z*-score > 2 and *p* < 0.05). Two activated transcription regulators were found: MYCN (*z* = 2.0) and NFE2L2 (*z* = 2.17). Furthermore, hypothetical activated and inhibited master regulators in derived causal networks were suggested by the analysis (Table 4).

### 2.5. Transcriptional Validation of Proteomic Data Using RT-Polymerase Chain Reaction (PCR) Assays

**Figure 7:**
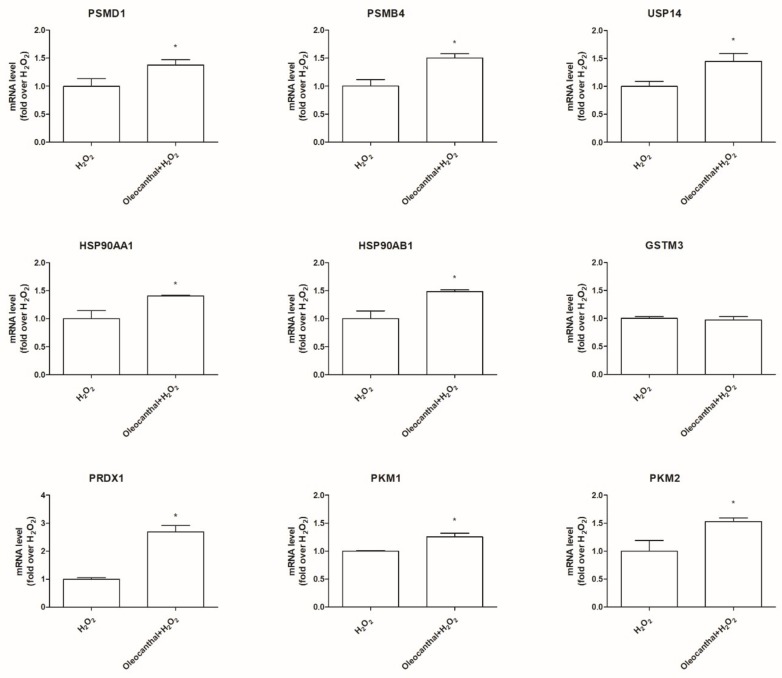
Transcriptional validation of proteomic data. Differentiated SH-SY5Y were pretreated with 10 µM oleocanthal for 24 h and then exposed to 700 µM H_2_O_2_ for 1 h. Total RNA was isolated, and the mRNA level of target genes was quantified using RT-PCR normalized to 18S rRNA reference gene as reported in Materials and Methods. Triplicate reactions were performed for each experiment. Each bar represents the mean ± SEM of three independent experiments. Data were analyzed by Student’s *t-*test. * *p* < 0.05 with respect to H_2_O_2_.

We selected 9 genes encoding significantly expressed proteins for RT-PCR validation: HSP90AA1, HSP90AB1 which encode for the isoforms α and β of the heat shock protein Hsp90; PSMD1, PSMB4, and USP14 which encode for proteasome 26S subunit non-ATPase 1 (Psmd1), proteasome subunit β 4 (Psmb4), and ubiquitin carboxyl-terminal hydrolase 14 (Usp14), which are related to the proteasome activity; GSTM3 and PRDX1, which encode for the antioxidant enzymes glutathione *S*-transferase mu 3 (Gstm3) and peroxiredoxin 1 (Prdx1); and PKM1 and PKM2, which encode for the isoforms 1 and 2 of the glycolytic enzyme pyruvate kinase (Pkm). As shown in Figure 7, except for Gstm3, whose expression was not modulated by oleocanthal in the presence of H_2_O_2_, all the other genes were up- or down-regulated in agreement with data reported in Table 2.

### 2.6. Role of Oleocanthal in the Modulation of the Ubiquitin–Proteasome System

**Figure 8:**
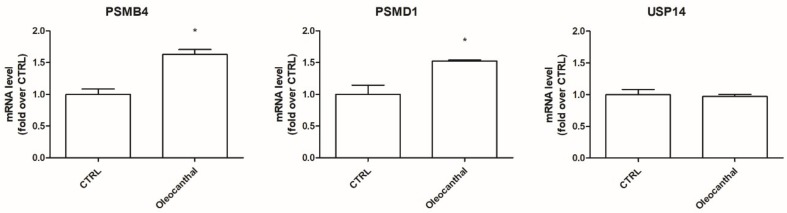
Effect of oleocanthal on the ubiquitin–proteasome system in the absence of H_2_O_2_. Differentiated SH-SY5Y were treated with 10 µM oleocanthal for 24 h. Total RNA was isolated, and the mRNA level of target genes was quantified using RT-PCR normalized to 18S rRNA reference gene as reported in Materials and Methods. Triplicate reactions were performed for each experiment. Each bar represents the mean ± SEM of three independent experiments. Data were analyzed by Student’s *t-*test. * *p* < 0.05 with respect to CTRL.

Proteomic and RT-PCR results demonstrated that oleocanthal in the presence of peroxide is able to up-regulate two proteasomal subunits with respect to H_2_O_2_, suggesting its positive role in the degradation of misfolded and oxidized proteins; on the other hand, it up-regulates the expression of the de-ubiquitinating enzyme Usp14 that reduces the number of proteins conveyed to the proteasome. To better investigate this conflicting point, we studied the effect of oleocanthal on these three proteins in the absence of oxidative stress by RT-PCR (Figure 8). Interestingly, in the absence of oxidative stress, oleocanthal up-regulated proteasome subunits Psmb4 and Psmd1 but did not influence Usp14 expression.

### 2.7. Involvement of HSP90 in Oleocanthal Protection against Oxidative Stress

**Figure 9:**
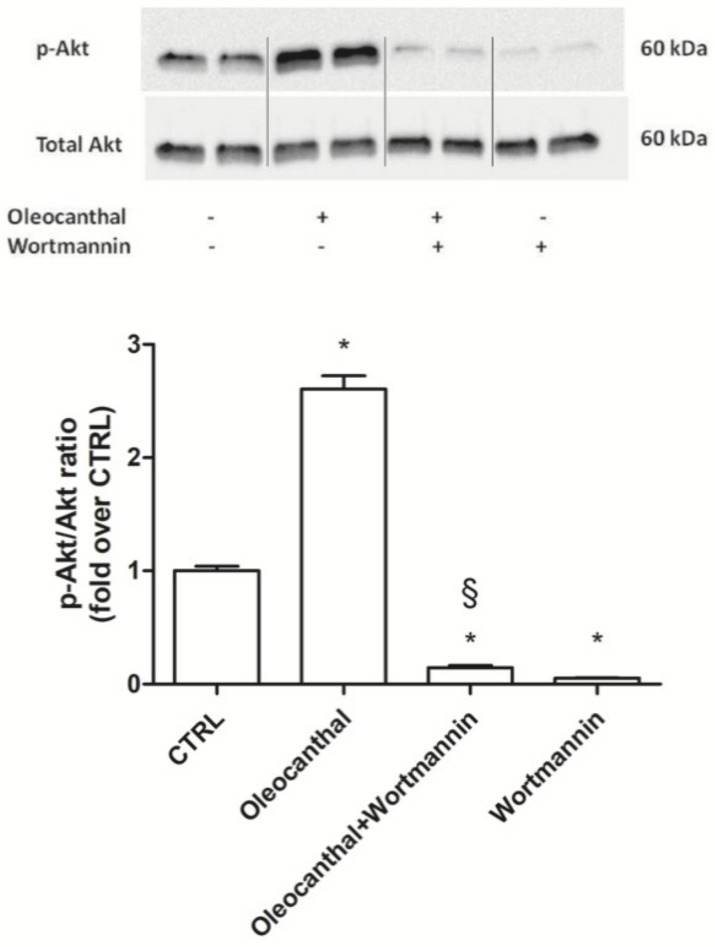
Akt kinase activation following oleocanthal treatment in the presence of PI3K-specific inhibitor. Differentiated SH-SY5Y were exposed to 100 nM wortmannin 1 h before 10 µM oleocanthal treatment for 1 h. Proteins were separated by SDS-PAGE and immunoblotted for total and phosphorylated form of Akt as reported in Materials and Methods. Each bar represents the mean ± SEM of three independent experiments. Data were analyzed by one-way ANOVA followed by Bonferroni’s test. * *p* < 0.05 with respect to CTRL; ^§^ *p* < 0.05 with respect to oleocanthal.

**Figure 10:**
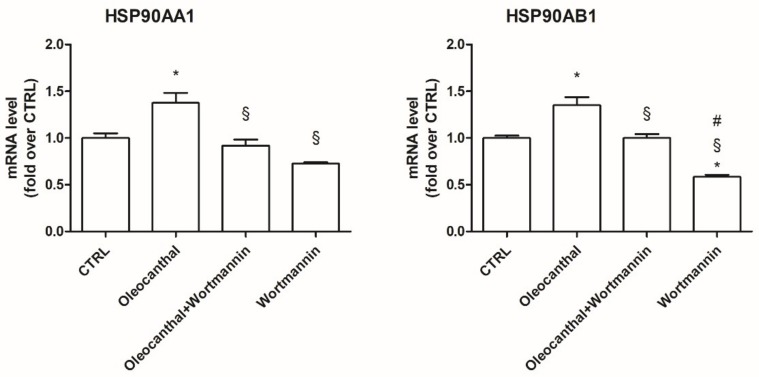
Akt-specific inhibitor wortmannin prevents oleocanthal-induced HSP90s. Differentiated SH-SY5Y were treated with 100 nM wortmannin 1 h before exposure to 10 µM oleocanthal for 24 h. Total RNA was isolated, and the mRNA level of target genes was quantified using RT-PCR normalized to 18S rRNA reference gene as reported in Materials and Methods. Triplicate reactions were performed for each experiment. Each bar represents the mean ± SEM of three independent experiments. Data were analyzed by one-way ANOVA followed by Bonferroni’s test. * *p* < 0.05 with respect to CTRL; ^§^ *p* < 0.05 with respect to oleocanthal; ^#^ *p* < 0.05 with respect to oleocanthal + wortmannin.

**Figure 11:**
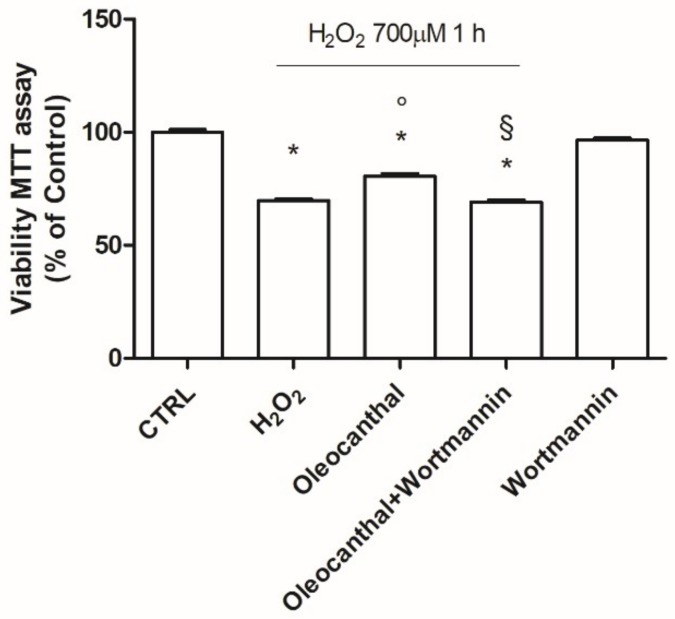
Effect of Akt inhibitor wortmannin on H_2_O_2_-induced injury in differentiated SH-SY5Y. Cells were treated with 10 µM oleocanthal for 24 h in absence/presence of 100 nM wortmannin prior to peroxide exposure, then cell viability was assessed with MTT assay. Each bar represents the mean ± SEM of three independent experiments. Data were analyzed by one-way ANOVA followed by Bonferroni’s test. * *p* < 0.05 with respect to CTRL; ° *p* < 0.05 with respect to H_2_O_2;_^§^ *p* < 0.05 with respect to oleocanthal.

As our proteomic and transcriptional data showed a marked and significant up-regulation of Hsp90α and β by oleocanthal in the presence of H_2_O_2_ with respect to H_2_O_2_ alone, we decided to investigate the role of these heat shock proteins in the observed oleocanthal protection against peroxide. A strict correlation between Akt activity and Hsp90 has been demonstrated [33], so we studied the effect of oleocanthal treatment on both Akt activation (phosphorylation) (Figure 9) and Hsp90α and β expression (Figure 10) in the absence/presence of wortmannin, which affects various signal transduction cascades by inhibiting phosphatidylinositol-3-kinases (PI3Ks), thereby blocking Akt phosphorylation. Interestingly, oleocanthal induced a strong activation of Akt (Figure 9). As expected, in the presence of wortmannin the phosphorylated form of Akt was quite undetectable. Oleocanthal treatment was able to significantly up-regulate both Hsp90 isoforms; meanwhile, wortmannin completely abrogated the up-regulation induced by oleocanthal, maintaining Hsp90α and β levels comparable to those in control cells. In order to clarify the involvement of these heat shock proteins in oleocanthal protection against oxidative stress, SH-SY5Y cells were treated with oleocanthal in the absence/presence of wortmannin and exposed to H_2_O_2_; cell viability was evaluated by MTT assay (Figure 11). Interestingly, in the presence of wortmannin, cell viability of oleocanthal-treated cells was comparable to that of H_2_O_2_-treated cells, suggesting that Hsp90 and/or Akt are essential to conferring neuroprotection against peroxide.

## 3. Discussion

In this study, we demonstrated that oleocanthal significantly protects differentiated human neuroblastoma SH-SY5Y cell line from oxidative stress induced by H_2_O_2_. In particular, oleocanthal reduces intracellular ROS levels and increases endogenous GSH levels. To further elucidate the mechanisms behind oleocanthal’s neuroprotective activity we used proteomic analysis that revealed an involvement of the ubiquitin–proteasome system, heat shock proteins, pyruvate kinases, and peroxiredoxin 1. Proteomic data were validated by real-time PCR analysis in order to have a complementary and wider vision of oleocanthal effect on both gene and protein expression. Since the levels of transcription of the selected genes were similar to their translation, we considered the comparable change in mRNA levels as a validation of proteomic data. A fundamental issue in the study of nutraceutical compounds is their bioavailability. The pharmacokinetics and bioavailability of oleocanthal have been poorly investigated. Under simulated gastric acid conditions, it has been shown that oleocanthal is stable in acidic conditions at 37 °C and almost half of the oleocanthal diffused from the oil phase into the aqueous solution [34]. Only one study has investigated the bioavailability of oleocanthal by identifying oleocanthal and its metabolites in urines, demonstrating oleocanthal metabolism in the human body [35]. Oleocanthal is mainly metabolized through phase I metabolism, like hydroxylation, hydrogenation, and hydration. Some of the hydrogenated oleocanthal metabolites are further metabolized through phase II metabolism [35]. Even if no studies directly examined the blood–brain barrier (BBB) permeability of oleocanthal, different studies indirectly demonstrated its ability to reach the brain. In particular, Qosa et al. reported a decreased amyloid load in the hippocampus of TgSwDI mice after oleocanthal treatment [21]; meanwhile, in C57BL/6 mice, Abuznait et al. demonstrated that oleocanthal induces P-gp and LRP1, which are responsible for Aβ clearance across the BBB, and increases Aβ degradation by up-regulating Aβ-degrading enzymes [25], strongly suggesting its ability to reach the brain.

The ubiquitin–proteasome system is the main proteolytic complex responsible for the removal of misfolded and damaged intracellular proteins, often produced upon oxidative stress [36]. The proteasome is a non-lysosomal threonine protease [37] that degrades both normal and damaged proteins into short peptides by the internal protease activities [38,39]. It has been observed that the impairment of the ubiquitin–proteasome system is implicated in the pathogenesis of many neurodegenerative diseases [40] like Alzheimer’s disease, Parkinson’s disease, Lewy body dementia, Pick disease, frontotemporal dementia, and Huntington’s disease (HD) [41]. On these bases, the induction of the proteasome system has been emerging as a therapeutic target to counteract these diseases. To our best knowledge, no studies have explored the effect of oleocanthal on the ubiquitin–proteasome system upon oxidative stress. Interestingly, our proteomic data, obtained in the presence of oxidative stress, indicate the up-regulation of two proteasome subunits, namely, the 26S proteasome non-ATPase regulatory subunit and the proteasome subunit β type-4, and the up-regulation of the ubiquitin carboxyl-terminal hydrolases 14 (Usp14). As Usp14 negatively regulates proteasome activity by ubiquitin chain disassembly as well as by a noncatalytic mechanism [42], oleocanthal, in the presence of oxidative stress, has two opposite effects: on one side, it up-regulates the proteasome and, on the other side, it up-regulates Usp14, abrogating the suggested enhancement of the proteasome activity. For this reason, we examined the effect of oleocanthal in the absence of oxidative stress by real-time PCR. Intriguingly, in normal conditions, oleocanthal is able to induce proteasome activity as it up-regulates proteasome subunits Psmd1 and Psmb4 and does not affect Usp14 expression, suggesting that oleocanthal could have a role in the degradation of misfolded proteins rather than in the degradation of oxidized ones. Our observations are in agreement with different studies that demonstrated that oleocanthal counteracts neurodegenerative diseases by enhancing the clearance of misfolded proteins like amyloid-β, phosphorylated tau, and α-synuclein [27,43,44].

Hsp90 is a chaperone protein important for maintaining stability, maturation, and signaling of Hsp90 client proteins [45]. It has been demonstrated that Hsp90 maintains cell proliferation and cell survival by different mechanisms. Hsp90 may inhibit apoptosis by binding to apoptotic peptidase-activating factor 1, resulting in the prevention of apoptosome formation, caspase activation, and apoptotic cell death [46]. In addition, Taiyab et al. showed that inhibition of Hsp90 results in ER stress-induced apoptosis in rat histiocytoma [47]. A strict correlation between Hsp90 and Akt has been demonstrated [33], as the inhibition of the binding of Akt to Hsp90 inactivates Akt and makes cells more susceptible to apoptosis-inducing stimuli [48]. Interestingly, Xie et al. observed that the inhibition of Akt activity led to a reduction of Hsp90 level, suggesting that Akt activation can elevate Hsp90 protein levels [49]. Our data demonstrated that oleocanthal, upon oxidative stress, is able to up-regulate Hsp90. As Hsp90 acts with Akt, we also evaluated the effect of oleocanthal treatment on Akt activation (phosphorylation). As expected, we measured a strong and significative activation of Akt. Moreover, Akt inhibition by wortmannin led to the inhibition of the up-regulation of Hsp90 induced by oleocanthal, in agreement with the previous observation by Xie et al. Of note, the fact that wortmannin totally abrogated oleocanthal protection against oxidative stress suggests a fundamental role of Akt and/or Hsp90 in the protective mechanisms elicited by oleocanthal against oxidative stress in SH-SY5Y neuronal cells. As wortmannin can affect other signaling cascades apart from Akt, we could not exclude that oleocanthal regulates neuroprotective effects through other signaling pathways. Further studies are necessary to fully elucidate this aspect.

Our proteomic and expression data indicated a significative up-regulation of Pkm1 and Pkm2 by oleocanthal in the presence of oxidative stress. Pyruvate kinase, catalyzing the final rate-limiting step in glycolysis, is a crucial enzyme for glucose metabolism and energy production in the brain. Elevated levels of pyruvate kinase have been suggested to be protective in neurodegeneration as decreased aerobic glycolysis in the brain leads to a loss of cell survival mechanisms that counter pathogenic processes underlying neurodegeneration [50]. Moreover, Luo et al. demonstrated that Pkm hinders the Aβ fibrillation and reduces the toxicity of Aβ aggregates in SH-SY5Y cells, suggesting that Pkm interferes with the stability of the Aβ oligomer by hydrophobic and hydrophilic interactions of its surface [51].

Oleocanthal also induced a strong up-regulation of the antioxidant enzyme Prdx1 in the presence of H_2_O_2_, as indicated by our proteomic and expression results. Peroxiredoxins play an important role in cell proliferation, redox signaling, differentiation, and gene expression [52,53]. In particular, Prdx1 eliminates hydrogen peroxide produced during cellular metabolism [54] and participates in cell survival by enhancing the expression of the pro-survival factor protein kinase B (PKB) [55]. The role of Prdx1 in AD has been recently highlighted by Majd et al. who observed reduced levels of Prdx1 and 2 in postmortem brains of AD [56]; meanwhile, Schreibelt et al. analyzed the functional role of Prdx1 using a brain endothelial cell line overexpressing Prdx1 and showed that enhanced Prdx1 expression in brain endothelial cells increased BBB integrity and reduced monocyte adhesion to and migration across a brain endothelial cell layer [57].

Focusing on proteomic results, the quantitative two-dimensional analysis showed that the insult induced by peroxide induced a significantly different expression of four Prdx in the human neuroblastoma SH-SY5Y cell line. Interestingly, two near spots, different for the pI value and for level of expression, were detected for PRDX1, 2, and 6 in our samples, suggesting the presence of post-translational modification. Post-translational modification and intracellular protein–protein interactions have the potential to influence Prdx catalytic activity and susceptibility to hyperoxidation [58]. We found for all three Prdx an increase of expression of more acidic spots and a significant reduction of more basic spots in the H_2_O_2_ sample with respect to the control. The protective effect of oleocanthal takes place by way of an increase of more acidic spots of Prx1. The nature of this modification needs to be investigated and underlines a mechanism of control of protein activity.

**Figure 12:**
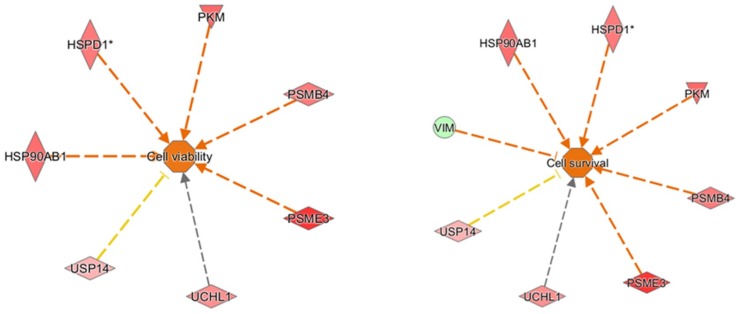
Downstream functions. Predicted activated functions based on up- (red) and down- (green) regulated proteins. Arrows predict a direct (orange) or indirect (grey) action on cell function. T bar predicts an inhibition on cell function. * This protein has been identified in many spots.

Finally, all proteins found differentially expressed from a comparison of oleocanthal + H_2_O_2_ vs. H_2_O_2_ were analyzed by IPA to explore in depth the network, functions, and molecules (e.g., upstream regulators) that could play a role in the protection induced by oleocanthal. The molecular functions defined by our differentially expressed proteins were well arranged in protein synthesis, cell death and survival, protein degradation, and cellular function and maintenance. On the other hand, the up-regulation of heat shock protein and proteasome members in addition to ubiquitin enzymes and pyruvate kinase concurred to predict cellular viability and vitality (Figure 12). As concerns upstream regulators, intriguing results arose from the IPA analysis. Along the list of noteworthy potential activated or inhibited upstreams were NFE2L2, HSF1, and ATF7.

NFE2L2, which encodes for the transcription factor Nrf2, is important for the coordinated up-regulation of genes in response to oxidative stress. Very recent studies showed neuroprotective actions [59,60] through the activation of Akt/Nrf2/antioxidant enzymes in neuronal cells. On the other hand, the significant up-regulation of the active form of Akt that we found in our results agrees with an activation state of Nrf2.

HSF1 works as a stress-inducible and DNA-binding transcription factor that plays a central role in the transcriptional activation of the heat shock response, leading to the expression of a large number of chaperone heat shock proteins. A central role of HSF1 in synaptic fidelity and memory consolidation has been recently suggested by Hooper and coworkers [61] who showed that the activation of HSF1 alone augmented vesicle transport and synaptic scaffolding proteins. Meanwhile, HSF1 agonists can improve cognitive function in dementia models and activation of neuroprotective signaling pathways. Finally, HSF1 protected neurons from death caused by accumulation of misfolded proteins. This neuroprotection result was abrogated by inhibition of classical deacetylases such as SIRT1 [62].

As concerns ATG7 (autophagy-related 7), a predicted inhibition was advanced by IPA analysis. ATG7 is an E1-like activating enzyme involved in the 2-ubiquitin-like systems required for cytoplasm-to-vacuole transport and autophagy. Recent findings reveal that selective neuronal deletion of ATG7 is strongly protective against neuronal death and overall brain hypoxic–ischemic injury [63].

Overall, our results, combined with IPA analysis, suggest that the major part of protein targets of oleocanthal in response to peroxide insult belongs to the proteostasis network including proteasome, ubiquitin, and chaperone proteins which move to restore equilibrium between protein synthesis, folding, trafficking, secretion, and degradation in different cell compartments. Moreover, our findings add new evidence on the effects of oleocanthal on heat shock protein 90, Pkm1 and 2, and Prdx1.

## 4. Materials and Methods

### 4.1. Materials

MTT, DCFH-DA, H_2_O_2_, dimethyl sulfoxide (DMSO), MCB, Phosphate-Buffered Saline (PBS), bovine serum albumin (BSA) Dulbecco’s modified Eagle’s medium (DMEM), fetal bovine serum (FBS), penicillin/streptomycin, wortmannin, primers listed in Table 5, mammalian protease inhibitor mixture, radioimmunoprecipitation assay (RIPA) buffer, all trans retinoic acid (RA), and all other chemicals of the highest analytical grade were purchased from Sigma Chemical (St. Louis, MO, USA). PhosSTOP was purchased from Roche Diagnostics (Mannheim, Germany).

### 4.2. Cell Culture and Treatments

The SH-SY5Y human neuroblastoma cell line was obtained from Sigma-Aldrich (Milan, Italy). Cells were grown in DMEM supplemented with 10% (*v*/*v*) of FBS, 2 mM glutamine, 50 U/mL of penicillin, and 50 μg/mL of streptomycin and maintained at 37 °C in a humidified incubator with 5% CO_2_ as previously reported [64]. Cell differentiation was induced, reducing serum levels of the medium to 1% with 10 µM of RA for seven days prior to treatments. Differentiated SH-SY5Y were treated with oleocanthal for 1 or 24 h. Oleocanthal was dissolved in DMSO and kept at −20 °C until use. The control group was treated with an equivalent volume of the vehicle alone. Oxidative stress was induced by exposing cells to 700 µM H_2_O_2_.

### 4.3. MTT Assay

Cells were treated with different concentrations of oleocanthal (1–10 µM) for 24 h prior to induction of oxidative stress. Cell viability was evaluated by measuring formazan formation as previously reported [64,65]. Briefly, after treatments, cells were incubated with 0.5 mg/mL of MTT solution for 1 h at 37 °C. After incubation, MTT was removed and 100 µL of DMSO were added, and the absorbance was recorded at λ = 595 nm using a microplate spectrophotometer (VICTOR3 V Multilabel Counter; PerkinElmer, Wellesley, MA, USA).

### 4.4. Intracellular ROS Production Assay

The production of intracellular reactive oxygen species was evaluated using the fluorescent probe DCFH-DA as reported in [66]. Briefly, differentiated SH-SY5Y were treated with oleocanthal for 24 h and then incubated with 10 µM DCFH-DA in DMEM *w*/*o* FBS for 30 min. After DCFH-DA removal, cells were incubated with H_2_O_2_ for 30 min. Cells were washed with PBS and the fluorescence was measured using 485 nm excitation and 535 nm emission with a microplate spectrofluorometer (VICTOR3 V Multilabel Counter, PerkinElmer, Wellesley, MA, USA).

### 4.5. Intracellular GSH Levels Assay

The levels of reduced glutathione (GSH) were evaluated by a fluorometric assay, using the fluorescent probe MCB [64,67]. Briefly, differentiated SH-SY5Y pretreated with oleocanthal were exposed to H_2_O_2_ for 1 h and incubated with 50 µM MCB in serum-free medium for 30 min at 37 °C. After incubation, cells were washed in PBS and the fluorescence was measured at 355 nm (excitation) and 460 nm (emission) with a microplate spectrofluorometer (VICTOR3 V Multilabel Counter; PerkinElmer, Wellesley, MA, USA).

### 4.6. RNA Extraction

Total RNA was extracted using an RNeasy Mini Kit (QIAGEN GmbH, Hilden, Germany), following the manufacturer’s protocol. The yield and purity of the RNA were measured using a NanoVue Spectrophotometer (GE Healthcare, Milano, Italy).

### 4.7. Analysis of mRNA Levels by Reverse Transcriptase Polymerase Chain Reaction

cDNA was obtained by reverse transcribing mRNA starting from 1 μg of total RNA using an iScript cDNA Synthesis Kit (BIO-RAD, Hercules, CA, USA), following the manufacturer’s protocol. The subsequent polymerase chain reaction (PCR) was performed in a total volume of 10 μL containing 2.5 μL (12.5 ng) of cDNA, 5 μL SsoAdvanced Universal SYBR Green Supermix (BIO-RAD), and 0.5 μL (500 nM) of each primer. The primers used are reported in Table 5; 18S rRNA was used as reference gene.

### 4.8. Western Blotting

After treatments, differentiated SH-SY5Y were collected and homogenized in RIPA buffer with a mammalian protease inhibitor mixture and PhosSTOP. Samples were boiled for 5 min prior to separation on 10% MiniPROTEAN TGX Precast Protein Gels (BIO-RAD, Hercules, CA, USA). The proteins were transferred to a nitrocellulose membrane (Hybond-C; GE Healthcare, Buckinghamshire, UK) in Tris-glycine buffer at 110 V for 90 min. Membranes were then incubated in a blocking buffer containing 5% (*w*/*v*) skimmed milk and incubated with anti-phospho-Akt, anti-Akt (Cell Signaling Technology, Beverly, MA, USA), and anti-β-actin (Sigma-Aldrich), which was used as internal normalizer, overnight at 4 °C on a three-dimensional rocking table. The results were visualized by chemiluminescence using Clarity Western Enhanced Chemiluminescence (ECL) reagent according to the manufacturer’s protocol (BIO-RAD). Semiquantitative analysis of specific immuno-labeled bands was performed using ImageLabTM 5.2 Software (BIO-RAD).

### 4.9. Sample Preparation for Proteomic Analysis

For proteomic analysis, differentiated SH-SY5Y cells (about 7 × 10^5^ cells) were treated with oleocanthal or H_2_O_2_, or with both for 24 h as described above. At the end of treatment, cells were collected and washed with PBS. After centrifugation (1000× *g* for 5 min), the resulting pellets were immediately frozen and stored at −80 °C until use. For proteomic studies, each condition was performed in triplicate. 

### 4.10. 2DE Analysis

Cell pellets were resuspended in rehydration solution [68], and protein contents of resulting protein extracts were measured with an RC-DC Protein Assay from Bio-Rad.

2DE was carried out as previously described [69]. Briefly, 200 µg of proteins were filled up to 450 μl in rehydration solution. Immobiline Dry-Strips (GE Health Care Europe; Uppsala, Sweden); 18 cm, nonlinear gradient pH 3–10) were rehydrated overnight in the sample and then transferred to the Ettan IPGphor Cup Loading Manifold (GE Healthcare) for isoelectrofocusing (IEF). The second dimension (Sodium Dodecyl Sulphate-Polyacrylamide Gel Electrophoresis; SDS-PAGE) was carried out by transferring the proteins to 12% polyacrylamide, running at 16 mA per gel and 10 °C for about 16 h, using the Protean^®^ Plus Dodeca Cell (Bio-Rad). The gels were stained with Ruthenium II tris (bathophenanthroline disulfonate) tetrasodium salt (SunaTech Inc., Suzhou, China) (RuBP). ImageQuant LAS4010 (GE Health Care) was used for the acquisition of images. The analysis of images was performed using Same Spot (v4.1, TotalLab, Newcastle Upon Tyne, UK) software. The spot volume ratios between the four different conditions were calculated using the average spot normalized volume of the three biological replicates performed in duplicate. The software included statistical analysis calculations.

### 4.11. 2DE Statistical Analysis

A comparison among the different treatments was performed. The significance of the differences of normalized volume for each spot was calculated by the software Same Spot including the analysis of variance (ANOVA test). The protein spots of interest were cut out from the gel and identified by LC-MS analysis.

### 4.12. Spot Digestion and Protein Identification

The gel pieces were washed twice with the wash buffer (25 mM NH_4_HCO_3_ in 50% acetonitrile (ACN)). Afterwards, proteins were reduced with 10 mM ditiothreitol (DTT) (45 min, 56 °C), and alkylated with 55 mM iodoacetamide (IAA) (30 min, RT, in the dark). After two washes with the wash buffer, spots were completely dried in a centrivap vacuum centrifuge. The dried pieces of gel were rehydrated for 30 min at 4 °C in 30 μL of trypsin porcine (Promega, Madison, WI, USA) solution (3 ng/μL in 100 mM NH_4_HCO_3_) and then incubated at 37 °C overnight. The reaction was stopped by adding 10% trifluoroacetic acid (TFA). Samples were analyzed by LC-MS as described [70] using a shorter chromatographic gradient and in autoMS mode. Each digested spot sample was analyzed by LC-MS/MS using a Proxeon EASY-nLCII (Thermo Fisher Scientific, Milan, Italy) chromatographic system coupled to a Maxis HD UHR-TOF (Bruker Daltonics GmbH, Bremen, Germany) mass spectrometer. Peptides were loaded on the EASY-Column TM C18 trapping column (2 cm L., 100 µm I.D, 5 µm ps, Thermo Fisher Scientific), and subsequently separated on an Acclaim PepMap100 C18 (75 µm I.D., 25 cm L, 5 µm ps, Thermo Fisher Scientific) nanoscale chromatographic column. The flow rate was set to 300 nL/min and the gradient was from 2% to 20% of B in 12 min followed by 20 to 45% in 9 min and from 45% to 90% in 2 min (total run time 35 min). Mobile phase A was 0.1% formic acid in H_2_O and mobile phase B was 0.1% formic acid in acetonitrile. The mass spectrometer, typically providing a 60,000 full width at half maximum (FMHW) resolution throughout the mass range, was equipped with a nanoESI spray source. The mass spectrometer was operated in Data Dependent Acquisition mode (DDA), using N_2_ as the collision gas for collision-induced dissociation (CID) fragmentation. Precursors in the range 400 to 2200 *m*/*z* (excluding 1220.0–1224.5 *m*/*z*) with a preferred charge state +2 to +5 and absolute intensity above 4706 counts were selected for fragmentation in a fixed cycle time of 3 s. After two spectra, the precursors were actively excluded from selection for 36 s. Isolation width and collision energy for MS/MS fragmentation were set according to the mass and charge state of the precursor ions (from 2+ to 5+ and from 21 to 55 eV). In-source reference lock mass (1221.9906 *m*/*z*) was acquired online throughout runs.

The raw data were processed using PEAKS Studio v7.5 software (Bioinformatic Solutions Inc., Waterloo, ON, Canada) using the function “correct precursor only”. The mass lists were searched against the nextprot database including isoforms (version as of June 2017; 42,151 entries) using 10 ppm and 0.05 Da as the highest error tolerances for parent and fragment ions, respectively. Carbamidomethylation of cysteines was selected as fixed modification and oxidation of methionines and deamidation of asparagine and glutamine as variable modifications allowing 2 missed cleavages.

### 4.13. Network Analysis

Proteins differentially expressed obtained from the oleocanthal + H_2_O_2_ vs. H_2_O_2_ comparison were functionally analyzed through the use of QIAGEN’s Ingenuity Pathway Analysis (IPA, QIAGEN Redwood City, CA, USA, www.qiagen.com/ingenuity) with the aim to determine the predominant canonical pathways and interaction network involved. Swiss-Prot accession numbers and official gene symbols were inserted into the software along with corresponding comparison ratios and *p* values. The network proteins associated with biological functions and/or diseases in the Ingenuity Pathways Knowledge Base were considered for the analysis. The created networks describe functional relationships among proteins based on known associations in the literature. A comparison of the different analyses was created and the upstream regulators whose activity appears to change in a significant manner according to the activation *z*-score value were shown. Finally, to generate plausible causal networks which explain observed expression changes, hidden connections in upstream regulators were also uncovered.

## Figures and Tables

**Table 1:** MS/MS data of protein spots differentially expressed grouped by functional class. MW, molecular weight; pI, isoelectric point; th, theoretical.

# Spot	Protein Name	Gene	ID	Peptide (n)	Unique	Coverage (%)	MW (th)	pI (th)
Metabolism								
381	Guanosine 5′-monophosphate synthase (glutamine-hydrolyzing)	GMPS	P49915	32	32	52	76,716	6.42
521	Pyruvate kinase PKM	PKM	P14618	36	6	59	57,937	7.96
559	Amidophosphoribosyltransferase	PPAT	Q06203	4	4	10	57,398	6.3
693	Gamma-enolase	ENO2	P09104	13	13	46	47,269	4.91
856	Cytosolic acyl coenzyme A thioester hydrolase	ACOT7	O00154	3	3	14	27,041	6.51
876	Alcohol dehydrogenase (NADP(+))	AKR1A1	P14550	14	14	50	36,573	6.32
939	Malate dehydrogenase, cytoplasmic	MDH1	P40925	7	7	32	36,426	6.91
950	ADP-sugar pyrophosphatase	NUDT5	Q9UKK9	6	6	26	24,328	4.87
1069	Enoyl-CoA hydratase, mitochondrial	ECHS1	P30084	13	13	43	31,387	8.34
1076	NADH:ubiquinone oxidoreductase iron-sulfur protein 3, mitocondrial	NDUFS3	O75489	8	8	32	30,242	6.98
1098	Triosephosphate isomerase	TPI1	P60174	12	12	57	26,669	6.45
2010	Phosphoglycerate kinase 1	PGK1	P00558	41	35	72	44,615	8.3
2010	Isocitrate dehydrogenasemitochondrial isoform2	IDH2	P48735	25	25	53	45,180	7.63
Protein synthesis, modification secretion folding								
296	Acylamino-acid-releasing enzyme	APEH	P13798	15	15	24	81,225	5.29
708	Adenosylhomocysteinase	AHCY	P23526	18	18	37	47,716	5.92
916	Small glutamine-rich tetratricopeptide repeat-containing protein alpha	SGTA	O43765	8	8	27	34,063	4.79
940	Ubiquitin thioesterase OTUB1	OTUB1	Q96FW1	5	5	23	31,284	4.85
1230	Protein DJ-1	PARK7	Q99497	11	11	49	19,891	6.32
1812	Platelet-activating factor acetylhydrolase IB subunit gamma	PAFAH1B3	Q15102	6	6	27	25,734	6.33
Protein degradation								
498	Ubiquitin carboxyl-terminal hydrolase 14	USP14	P54578	13	13	35	52,386	5.61
995	Proteasome activator complex subunit 3	PSME3	P61289	10	10	39	29,506	5.69
1111	Ubiquitin carboxyl-terminal hydrolase isozyme L1	UCHL1	P09936	29	29	81	24,824	5.33
Cytoskeleton, vesicle motility, transport, vesicle release								
430	Dihydropyrimidinase-related protein 3	DPYSL3	Q14195	29	24	57	73,911	5.94
436	Dihydropyrimidinase-related protein 3	DPYSL3	Q14195	30	30	58	73,911	5.94
441	Dihydropyrimidinase-related protein 3	DPYSL3	Q14195	29	24	58	73,911	5.94
1079	Ran-specific GTPase-activating protein	RANBP1	P43487	9	9	50	23,310	5.19
1425	Cofilin-1	CFL1	P23528	8	8	51	18,502	8.22
1743	F-actin-capping protein subunit β	CAPZB	P47756	17	17	56	30,629	5.69
RNA synthesis/metabolism								
590	ATP-dependent RNA helicase DDX39A	DDX39A	O00148	16	16	35	49,130	5.46
621	Spliceosome RNA helicase DDX39B	DDX39B	Q13838	18	18	39	48,991	5.44
624	Heterogeneous nuclear ribonucleoprotein H	HNRNPH1	P31943	25	13	55	49,229	5.89
836	Heterogeneous nuclear ribonucleoprotein A3	HNRNPA3	P51991	24	23	49	39,595	9.1
876	Heterogeneous nuclear ribonucleoprotein H3	HNRNPH3	P31942	12	12	58	36,926	6.37
915	Heterogeneous nuclear ribonucleoprotein H3	HNRNPH3	P31942	8	8	40	35,239	6.36
1735	Heterogeneous nuclear ribonucleoprotein A1	HNRNPA1	P09651	15	11	63	29,386	9.19
Chromatin remodeling and histone								
498	Poly(U)-binding-splicing factor PUF60 isoforma1	PUF60	Q9UHX1	10	10	28	59,875	5.19
Stress response								
181	26S proteasome non-ATPase regulatory subunit 1	PSMD1	Q99460	16	16	27	10,2258	5.14
334	Heat shock protein HSP 90-α	HSP90AA1	P07900	12	6	19	84,660	4.94
335	Heat shock protein HSP 90-β	HSP90AB1	P08238	15	8	24	83,264	4.96
498	60 kDa heat shock protein. mitochondrial	HSPD1	P10809	31	31	54	61,055	5.7
506	60 kDa heat shock protein. mitochondrial	HSPD1	P10809	54	54	73	61,055	5.7
1153	60 kDa heat shock protein. mitochondrial	HSPD1	P10809	4	4		61,055	5.7
701	DnaJ homolog subfamily A member 2	DNAJA2	O60884	15	15	35	45,746	6.06
995	Proteasome activator complex subunit 3	PSME3	P61289	10	10	39	29,506	5.69
1087	Peroxiredoxin-6	PRDX6	P30041	21	21	65	25,035	6
1090	Peroxiredoxin-6	PRDX6	P30041	20	20	67	25,035	6
1122	Heat shock protein β-1	HSPB1	P04792	21	21	77	22,783	5.98
1253	Peroxiredoxin-2	PRDX2	P32119	30	30	59	21,892	5.66
1269	Peroxiredoxin-2	PRDX2	P32119	20	20	59	21,892	5.66
1725	Peroxiredoxin-4	PRDX4	Q13162	4	4	17	30,540	5.86
1731	Peroxiredoxin-1	PRDX1	Q06830	21	21	72	22,110	8.27
1736	Peroxiredoxin-1	PRDX1	Q06830	17	17	72	22,110	8.27
1741	Thioredoxin-dependent peroxide reductase. mitochondrial	PRDX3	P30048	11	11	46	27,693	7.68
1743	F-actin-capping protein subunit β	CAPZB	P47756	17	17	56	30,629	5.69
Miscellaneous								
498	Ubiquitin carboxyl-terminal hydrolase 14	USP14	P54578	13	13	35	52,386	5.61
715	Programmed cell death protein 2-like	PDCD2L	Q9BRP1	3	3	13	39,417	4.71
826	Guanine nucleotide-binding protein G(i) subunit α-2	GNAI2	P04899	16	13	50	40,451	5.34
843	V-type proton ATPase subunit d 1	ATP6V0D1	P61421	5	5	20	40,329	4.89
843	Nucleophosmin	NPM1	P06748	14	14	52	32,575	4.64
950	Annexin A5	ANXA5	P08758	6	6	18	35,937	4.93
1153	High mobility group protein B1	HMGB1	P09429	4	4	24	24,894	5.6
1733	Chloride intracellular channel protein 1	CLIC1	O00299	16	15	61	26,923	5.09
1743	N(G).N(G)-dimethylarginine dimethylaminohydrolase 2	DDAH2	O95865	13	13	54	29,644	5.66
1767	MICOS complex subunit MIC60	IMMT	Q16891	41	41	60	82625	6.15
1813	28 kDa heat- and acid-stable phosphoprotein	PDAP1	Q13442	3	3	13	20,630	8.84

**Table 2:** List of differentially expressed proteins after comparison between H_2_O_2_ vs. Control (ctrl). ID: SwissProt accession Number; OD: Normalized optical density.

# Spot	ID	Gene	Protein Name	Ratio (OD) H_2_O_2_/Ctrl	*p*-Value
181	Q99460	PSMD1	26S proteasome non-ATPase regulatory subunit 1	0.76	0.026
335	P08238	HSP90AB1	Heat shock protein HSP 90-beta	0.67	0.018
381	P49915	GMPS	GMP synthase [glutamine-hydrolyzing]	0.75	0.014
430	Q14195	DPYSL3	Dihydropyrimidinase-related protein 3	0.58	0.002
436	Q14195	DPYSL3	Dihydropyrimidinase-related protein 3	0.74	0.001
441	Q14195	DPYSL3	Dihydropyrimidinase-related protein 3	1.56	0.022
506	P10809	HSPD1	60 kDa heat shock protein. mitochondrial	0.83	0.011
559	Q06203	PPAT	Amidophosphoribosyltransferase	0.57	0.046
621	Q13838	DDX39B	Spliceosome RNA helicase DDX39B	0.83	0.022
624	P31943	HNRNPH1	Heterogeneous nuclear ribonucleoprotein H	0.81	0.039
701	DNAJA2	O60884	DnaJ homolog subfamily A member 2	0.77	0.017
708	P23526	AHCY	Adenosylhomocysteinase	0.82	0.012
856	O00154	ACOT7	Cytosolic acyl coenzyme A thioester hydrolase	0.76	0.036
876	P14550	AKR1A1	Alcohol dehydrogenase (NADP(+))	0.69	0.043
876	P31942	HNRNPH3	Heterogeneous nuclear ribonucleoprotein H3		
915	P31942	HNRNPH3	Heterogeneous nuclear ribonucleoprotein H3	0.71	0.026
939	P40925	MDH1	Malate dehydrogenase. cytoplasmic	0.68	0.026
940	Q96FW1	OTUB1	Ubiquitin thioesterase OTUB1	0.73	0.017
950	P08758	ANXA5	Annexin A5	0.78	0.012
950	Q9UKK9	NUDT5	ADP-sugar pyrophosphatase		
1069	P30084	ECHS1	Enoyl-CoA hydratase. mitochondrial	0.78	0.023
1079	P43487	RANBP1	Ran-specific GTPase-activating protein	0.80	0.035
1087	P30041	PRDX6	Peroxiredoxin-6	5.84	0.007
1090	P30041	PRDX6	Peroxiredoxin-6	0.53	0.032
1098	P60174	TPI1	Triosephosphate isomerase	0.68	0.004
1122	P04792	HSPB1	Heat shock protein β-1	0.69	0.015
1179	P28070	PSMB4	Proteasome subunit β type-4	0.80	0.036
1253	P32119	PRDX2	Peroxiredoxin-2	0.17	4.4 × 10^−7^
1269	P32119	PRDX2	Peroxiredoxin-2	2.27	3.08 × 10^−5^
1725	Q13162	PRDX4	Peroxiredoxin-4	0.62	0.016
1731	Q06830	PRDX1	Peroxiredoxin-1	0.33	4.28 × 10^−6^
1736	Q06830	PRDX1	Peroxiredoxin-1	3.37	1.37 × 10^−6^
1741	P30048	PRDX3	Thioredoxin-dependent peroxide reductase mitochondrial	0.48	0.00041
1743	P47756	CAPZB	F-actin-capping protein subunit β	0.80	0.042
1743	O95865	DDAH2	N(G).N(G)-dimethylarginine dimethylaminohydrolase 2		
1767	Q16891	IMMT	MICOS complex subunit MIC60	0.72	0.021
1812	Q15102	PAFAH1B3	Platelet-activating factor acetylhydrolase IB subunit γ	0.76	0.021

**Table 3:** List of differentially expressed proteins after comparison between oleocanthal (OC) + H_2_O_2_ vs. H_2_O_2_. ID: SwissProt accession. Number; OD: Normalized optical density.

# Spot	ID	Gene	Protein Name	Ratio M ± SD (OD) OC + H_2_O_2_/H_2_O_2_	*p*-Value
181	Q99460	PSMD1	26S proteasome non-ATPase regulatory subunit 1	1.29	0.034
296	P13798	APEH	Acylamino-acid-releasing enzyme	1.28	0.009
334	P08238	HSP90AB1	Heat shock protein HSP 90-β	1.33	0.026
334	P07900	HSP90AA1	Heat shock protein HSP 90-alpha		
498	P54578	USP14	Ubiquitin carboxyl-terminal hydrolase 14	1.30	0.023
498	Q9UHX1	PUF60	Poly(U)-binding-splicing factor PUF60 isoforma1		
521	P14618	PKM	Pyruvate kinase PKM	1.35	0.035
590	O00148	DDX39A	ATP-dependent RNA helicase DDX39A	1.36	0.018
621	Q13838	DDX39B	Spliceosome RNA helicase DDX39B	1.29	0.013
715	Q9BRP1	PDCD2L	Programmed cell death protein 2-like	0.77	0.013
836	P51991	HNRNPA3	Heterogeneous nuclear ribonucleoprotein A3	0.64	0.023
843	P61421	ATP6V0D1	V-type proton ATPase subunit d 1	0.64	0.019
843	P06748	NPM1	Nucleophosmin		
940	Q96FW1	OTUB1	Ubiquitin thioesterase OTUB1	1.45	0.036
1109	P21266	GSTM3	Glutathione *S*-transferase Mu 3	1.23	0.036
1111	P09936	UCHL1	Ubiquitin carboxyl-terminal hydrolase isozyme L1	1.25	0.002
1153	P09429	HMGB1	High mobility group protein B1	0.43	0.049
1179	P28070	PSMB4	Proteasome subunit β type-4	1.30	0.024
1736	Q06830	PRDX1	Peroxiredoxin-1	1.21	0.029

**Table 4:** Master regulators predicted by IPA analysis.

Master Regulator	Molecule Type	Predicted Activation State	Activation *z*-Score	*p*-Value
GPR68	G-protein complex	Inhibited	−2.12	1.09 × 10^−4^
MARK2	Kinase	Inhibited	−2.33	2.67 × 10^−4^
miR-149-5p	Mature microRNA	Inhibited	−2.53	1.36 × 10^−4^
ATG7	Enzyme	Inhibited	−2.65	7.88 × 10^−6^
Smad2/3-Smad4	Complex	Inhibited	−2.83	3.02 × 10^−4^
PDE2A	Enzyme	Activated	+2.53	4.07 × 10^−5^
ELMO1	Other	Activated	+2.53	8.06 × 10^−5^
DOCK5	Other	Activated	+2.53	8.14 × 10^−5^
FARP2	Other	Activated	+2.53	1.07 × 10^−4^
ARHGEF6/7	Group	Activated	+2.53	1.07 × 10^−5^
BAIAP2	Kinase	Activated	+2.33	2.44 × 10^−4^
RAC1	Enzyme	Activated	+2.33	2.47 × 10^−4^
Hif	Complex	Activated	+2.33	3.28 × 10^−4^
ADORA1	G-protein complex	Activated	+2.33	4.12 × 10^−4^
NOX4	Enzyme	Activated	+2.31	3.59 × 10^−6^
NFE2L2	Transcription factor	Activated	+2.23	1.07 × 10^−5^
MYCN	Transcription factor	Activated	+2.00	3.85 × 10^−5^
HSF1	Transcription factor	Activated	+2.00	1.33 × 10^−3^

**Table 5:** List of primers for real-time PCR.

Gene	5′-Forward-3′	5′-Reverse-3′
*GSTM3*	TTGAGGCTTTGGAGAAAATC	TGAAAAGAGCAAAGCAAGAG
*HSP90AA1*	ATATCACAGGTGAGACCAAG	GTGACTGACACTAAAGTCTTC
*HSP90AB1*	TCTATTACATCACTGGTGAGAG	CTCTTCCCATCAAATTCCTTG
*PSMB4*	ACGCTGATTTCCAGTATTTG	CCATGAATGAATAGCTCTAGG
*PSMD1*	CCAGTTATTGGATAACCCAG	CTCCAATAGAGAGTGGTTTG
*USP14*	AGCCCTTAGAGATTTGTTTG	ATCCTGTTGAAGATACTGTCC
*PKM1*	GAGGCAGCCATGTTCCAC	TGCCAGACTCCGTCAGAACT
*PKM2*	CAGAGGCTGCCATCTACCAC	CCAGACTTGGTGAGGACGAT
*PRDX1*	GGGTCAATACACCTAAGAAAC	CTTCATCAGCCTTTAAGACC
*18S rRNA*	CAGAAGGATGTAAAGGATGG	TATTTCTTCTTGGACACACC

## Abbreviations

2DE	Two-dimensional electrophoresis
AD	Alzheimer’s disease
ANOVA	Analysis of variance
BBB	Blood–brain barrier
BSA	Bovine serum albumin
CID	Collision-induced dissociation
DCFH-DA	2,7-Dichlorodihydrofluorescein diacetate
DDA	Data dependent acquisition mode
DMEM	Dulbecco’s modified Eagle’s medium
DMSO	Dimethyl sulfoxide
DTT	Ditiothreitol
ECL	Enhanced chemiluminescence
FMHW	Full width at half maximum
GSH	Reduced glutathione
Gstm3	Glutathione *S*-transferase mu 3
IEF	Isoelectrofocusing
IPA	Ingenuity pathways aanalysis
MCB	Monochlorobimane
MTT	3-(4,5-Dimethylthiazol-2-yl)-2,5diphenyl-tetrazolium bromide)
NOX	Nicotinamide adenine dinucleotide phosphate oxidase
PBS	Phosphate-buffered saline
PCR	Polymerase chain reaction
PD	Parkinson’s disease
Prdx	Peroxiredoxin
Prk	Pyruvate kinase
Psmb4	Proteasome subunit β 4
Psmd1	Proteasome 26S subunit non-ATPase 1
RA	All trans retinoic acid
RIPA	Radioimmunoprecipitation assay
ROS	Reactive oxygen species
SDS-PAGE	Sodium dodecyl sulfate polyacrylamide gel electrophoresis
Usp14	Ubiquitin carboxyl-terminal hydrolase 14
